# The Association between Virus Prevalence and Intercolonial Aggression Levels in the Yellow Crazy Ant, *Anoplolepis Gracilipes* (Jerdon)

**DOI:** 10.3390/insects10120436

**Published:** 2019-12-04

**Authors:** Hung-Wei Hsu, Ming-Chung Chiu, Ching-Chen Lee, Chow-Yang Lee, Chin-Cheng Scotty Yang

**Affiliations:** 1 Laboratory of Insect Ecology, Graduate School of Agriculture, Kyoto University, Kyoto 606-8502, Japan; max7297@gmail.com; 2 Department of Biology, Graduate School of Science, Kobe University, Kobe 657-8501, Japan; b93612019@ntu.edu.tw; 3 Center for Ecology and Environment, Department of Life Science, Tunghai University, Taichung 40704, Taiwan; cclee86@thu.edu.tw; 4 Department of Entomology, University of California, Riverside, 900 University Avenue, Riverside, CA 92521, USA; chowyang.lee@ucr.edu; 5 Research Institute for Sustainable Humanosphere, Kyoto University, Gokasho, Uji, Kyoto 611-0011, Japan

**Keywords:** aggressive behavior, *Anoplolepis gracilipes*, colony structure, viral prevalence, yellow crazy ant

## Abstract

The recent discovery of multiple viruses in ants, along with the widespread infection of their hosts across geographic ranges, provides an excellent opportunity to test whether viral prevalence in the field is associated with the complexity of social interactions in the ant population. In this study, we examined whether the association exists between the field prevalence of a virus and the intercolonial aggression of its ant host, using the yellow crazy ant (*Anoplolepis gracilipes*) and its natural viral pathogen (TR44839 virus) as a model system. We delimitated the colony boundary and composition of *A*. *gracilipes* in a total of 12 study sites in Japan (Okinawa), Taiwan, and Malaysia (Penang), through intercolonial aggression assay. The spatial distribution and prevalence level of the virus was then mapped for each site. The virus occurred at a high prevalence in the surveyed colonies of Okinawa and Taiwan (100% infection rate across all sites), whereas virus prevalence was variable (30%–100%) or none (0%) at the sites in Penang. Coincidentally, colonies in Okinawa and Taiwan displayed a weak intercolonial boundary, as aggression between colonies is generally low or moderate. Contrastingly, sites in Penang were found to harbor a high proportion of mutually aggressive colonies, a pattern potentially indicative of complex colony composition. Our statistical analyses further confirmed the observed correlation, implying that intercolonial interactions likely contribute as one of the effective facilitators of/barriers to virus prevalence in the field population of this ant species.

## 1. Introduction

A number of traits, such as shifts in social structure, inbreeding tolerance, and adaptability to human-disturbed habitats, are disproportionally presented in invasive ants [1,2,3]. Among these traits, supercolonies—where workers work in physically separated colonies, and exhibit limited aggression towards each other but maintain high aggression to those from other supercolonies—are believed to be associated with the ecological success of a subset of invasive ants such as the Argentine ant (*Linepithema humile* [Mayr]), the big-headed ant (*Pheidole megacephala* [Fabricius]), and the yellow crazy ant (*Anoplolepis gracilipes* [Jerdon]) [4,5,6]. However, the absence of visible intercolonial boundaries may also facilitate the transmission of pathogens among colonies in the same supercolony, causing supercolony-wide epidemics if no disinfection mechanisms exist or the pathogen, with limited fitness, costs to their host [7]. The “vulnerable supercolony hypothesis” was proposed by Ugelvig and Cremer [8] and has been empirically tested by Tragust et al. In [7], supercolony structure was shown to be more susceptible to fungal infections due to the high frequency of intercolonial interactions in the invasive garden ant, *Lasius neglectus*. Furthermore, such a pattern is likely more profound for viral pathogens because viruses are generally more readily transmitted horizontally than other pathogen groups [9,10,11,12]. Consequently, one would predict that a population comprised of mutually tolerant colonies likely has a higher level of viral prevalence as a result of intensive intercolonial interactions, while variations of prevalence should be registered in a population with mixed supercolonial groups (i.e., aggression is only observed when workers are from different supercolonies). To assess such transmission dynamics, a critical step is to understand if intercolonial interactions serve as a potential transmission pathway of a given virus.

Native to the Paleotropics (but see [13]), the yellow crazy ant, *A*. *gracilipes,* was introduced into many islands in the Indian and Pacific Oceans and had destructive impacts on the native ecosystems on these islands [14,15]. Despite the presence of supercolonies in most of these island populations, previous studies have shown that populations of *A*. *gracilipes* in Christmas Island [14] and Taiwan (Lee, unpublished data) generally comprise of colonies that display limited aggression toward each other, while those in Penang, Malaysia, harbor a high proportion of mutually aggressive colonies [16]. These behavioral observations suggest that population structure may vary across where this ant is distributed. Coupled with the recent discovery of the TR44839 virus in the yellow crazy ant [17], such differences in intercolonial aggression behavior provide an excellent opportunity to test whether viral prevalence is associated with the social interactions of populations. The TR44839 virus was discovered in a confirmed invasive population of *A*. *gracilipes* in Australia and appears to be a member of the *Dicistroviridae* viral family based on phylogenetic analysis [17]. Despite no detailed fundamental information reported from the same study [17], viruses in this family are generally characterized as positive-sense, single-stranded RNA genomes, and usually persist in hosts asymptomatically, yet sometimes cause illness in their arthropod hosts [18].

Colony organization and interaction network play a critical role in regulating disease transmission dynamics in social insects. A majority of studies, however, focused on an intracolony level, and the potential effects from higher hierarchical levels (e.g., intercolony), are rarely tested (but see [7]). In this study, we hypothesized that viral prevalence is lower (or more variable) in populations of *A*. *gracilipes* characterized primarily by mutually aggressive colonies than those with colonies displaying none or limited aggression level. If such an association holds, the aggression between colonies is likely correlated with the viral prevalence. We first mapped the viral distribution among the colonies of *A*. *gracilipes* in numerous sites on three Asian islands (Okinawa, Japan; Taiwan; Penang, Malaysia) and assayed aggressive interactions between colonies at both within-site and between-site levels. We then examined if a correlation exists between the two variables in *A*. *gracilipes*. We believe that this is the first study that dissects the interplay between social interactions and viral prevalence in *A*. *gracilipes*, and offers critical information that would assist in developing an efficient virus-based pest management strategy.

## 2. Materials and Methods

### 2.1. Virus Detection and Confirmation of Viral Replication

**Figure 1:**
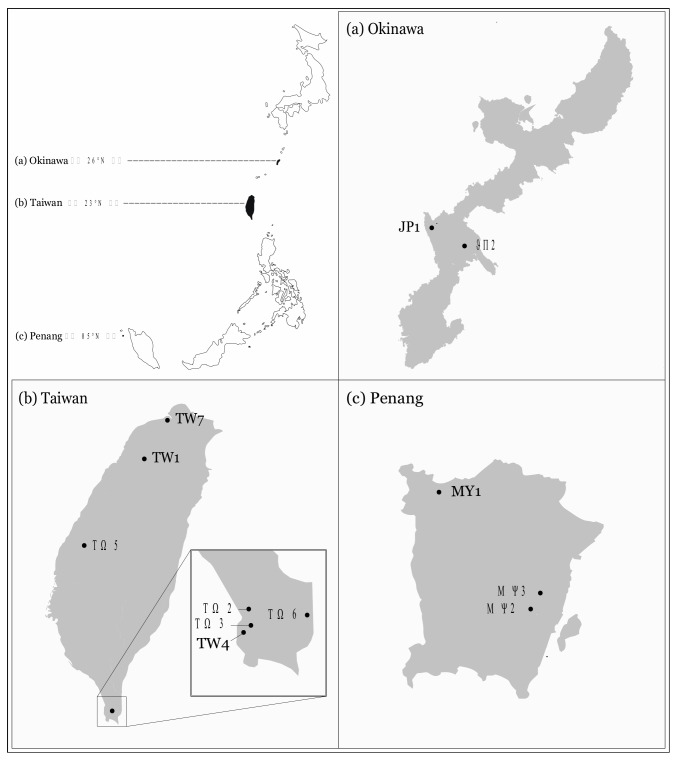
Geographic distribution of our study sites in (**a**) Okinawa (JP1 and JP2), (**b**) Taiwan (TW1-TW7), and (**c**) Penang (MY1-MY3).

We selected a total of 12 sites from three islands of varying latitudes, namely Okinawa, Japan (N = 2, JP1 & 2), Taiwan (N = 7, TW1-7) and Penang, Malaysia (N = 3, MY1-3), and collected various numbers of *A*. *gracilipes* colonies in each site (Figure 1). The colony in the current study was defined as a physical nest unit, containing all castes/stages (queens, workers and brood, and sometimes alates), clearly delineated from another unit by at least 100 m. A total of 28, 27 and 40 colonies (all sites combined) were collected from Okinawa, Taiwan, and Penang, respectively. We screened for the presence of the TR44839 virus in each site and calculated the infection rate based on the number of infected colonies over the total number of collected colonies in a site. To understand if the TR44839 virus can be horizontally transmitted among colonies, and also if the virus actively replicates in *A*. *gracilipes*, an artificial inoculation assay of the virus was carried out. Additionally, three colonies confirmed to be TR44839 virus-uninfected (note, these three colonies were not involved in subsequent aggression assay) were collected and fed with a fixed amount of 10% honey solution homogenized with virus-infected *A*. *gracilipes* workers for 24 h. Given the fact that the replication of the virus in the *Dicistroviridae* family proceeds via the synthesis of a complementary negative strand, the presence of a negative strand would serve as evidence of replication. We, therefore, attempted to detect the negative strand of the virus using strand-specific RT-PCR at Day 7 after feeding. The RNA of five workers from each colony was extracted and used as the template for cDNA synthesis, using oligo-dT primer and the strand-specific primer. The resultant cDNA was then added to the subsequent PCR reaction in which the target viral fragment was amplified using the previously reported primer set (TR44839: AGACGAACGAACAAAGGACCT / CAATTCAGTTCTGCGGGTGC) [17]. The PCR amplification protocol included 94 °C for 3 min, then 35 cycles of 94 °C for 30 s, 60 °C for 30 s, and 72 °C for 30 s, with a final extension step of 72 °C for 5 min. Samples were confirmed TR44839-virus-positive if a 130 bp fragment was visualized on the agarose gel electrophoresis. We included a positive control (RNA extracted from individuals previously confirmed positive by both gel electrophoresis and sequencing), negative control (RNA extracted from individuals previously confirmed negative for the virus), and blank (ddH_2_O) in every batch of RT-PCR reaction.

### 2.2. Aggression Assay

**Figure 2:**
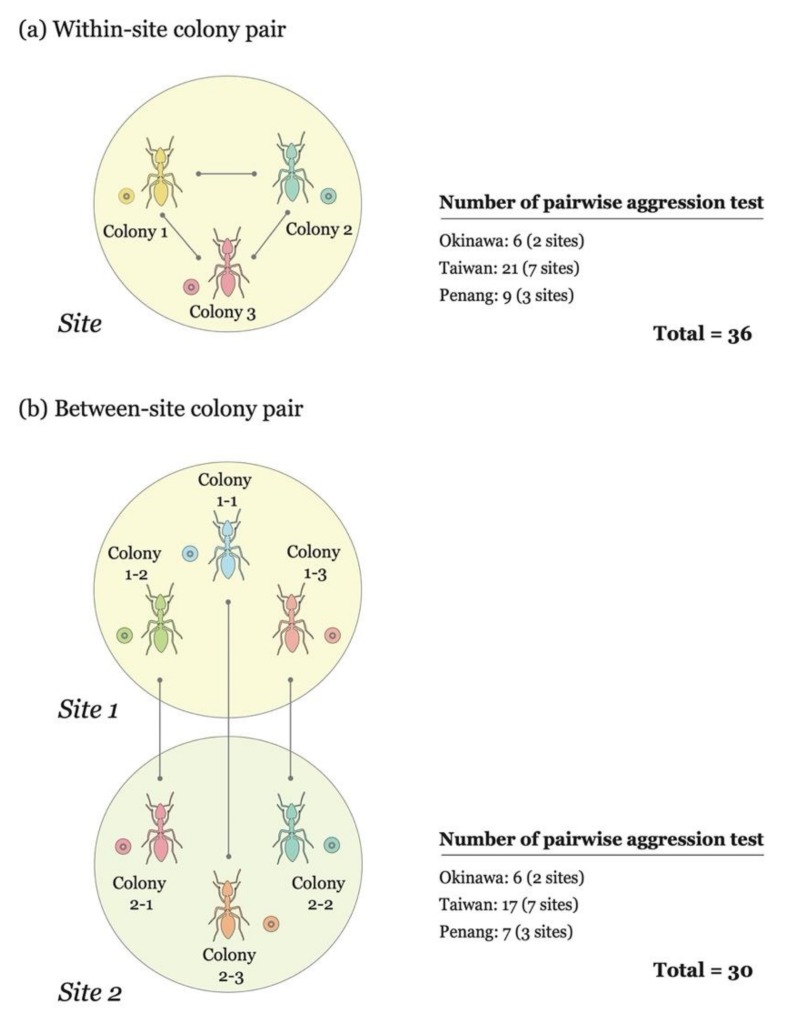
Graphic summary for within-site (**a**) and between-site (**b**) aggression assays. Colony pairs in the within-site aggression assay involve two colonies collected from the same site, while colony pairs involved in the between-site aggression assay are those collected from different sites of the same island. The number of pairwise aggression tests is indicated.

To assess the pattern of within-site intercolonial aggression in *A*. *gracilipes*, we randomly selected three colonies from each site and assayed aggression between workers from different colonies within the same site, resulting in 6, 21 and 9 pairwise aggression tests in Okinawa, Taiwan and Penang, respectively (Figure 2a; Supplementary Table S1). The aggression assay was carried out within a week of the field collection. An individual worker sampled from each of two colonies was placed in a 6 cm diameter petri dish with fluon-coated inner wall surface for 5 min. Each individual was tested only once and discarded. The behavioral interactions between two workers during a 5 min period were observed and scored as follows: 1 for ignoring or touching (antennation); 2 for avoidance; 3 for aggression (lunging, biting, or pulling); 4 for fighting (prolonged aggression) [19]. Ten replicates (ten individual workers) were performed for each colony pair, and aggression levels were averaged (Supplementary Table S1). Aggression assays were scored when the maximum level of aggression was achieved by two workers during each trial. For example, the observation was terminated if level 4 had been achieved before the end of the 5 min period. To understand the differences in overall intercolonial aggression levels between the three islands, aggression levels derived from all colony pairs, involving two colonies collected from the same island, were pooled and their significance analyzed using the Kruskal–Wallis rank-sum test, and the pairwise comparison analyzed using the Wilcoxon rank-sum test with Bonferroni correction.

### 2.3. Correlation between Infection Status and Aggressive Behavior

Intercolonial relationships (e.g., colony interactions) generally play an important role in shaping the efficiency of disease transmissions in social insects [20,21]. For example, high aggression between individual workers from different colonies is believed to decrease pathogen transmissions due to the limited contact and/or high causality and mortality following hostile interactions [22]. We, therefore, aimed to test the pathogen infection patterns associated with intercolonial aggression by analyzing the correlation between infection status and aggressive level. Additional aggression assay was carried out on colony pairs involving colonies from different sites of the same island (hereafter referred to as between-site colony pair, n = 30; Figure 2b; Supplementary Table S1), and the data were combined with that of the within-site colony pairs to ensure the dataset is statistically robust. Not all between-site colony pairs were subjected to the aggression test, particularly those from Taiwan, due to different field collection schemes. We then categorized all the colony pairs into three groups, based on the infection status of the two colonies involved, those groups being uninfected vs. uninfected (−/−), infected vs. uninfected (−/+), and infected vs. infected (+/+). In addition, the Kruskal–Wallis rank-sum test, and the pairwise comparisons using the Wilcoxon rank-sum test with Bonferroni correction, were used to determine whether aggression levels are associated with the infection status. We conducted the non-parametric hypothesis tests and data visualization with the basic function, fitted the linear mixed model using the lme4 package, and selected the best fitted model by MuMIn package in *R* software (version 3.5.2, *R* Core Team, 2018) [23].

## 3. Results

### 3.1. Horizontal Transimision, Replication and Prevalence of the TR44839 Virus

**Figure 3:**
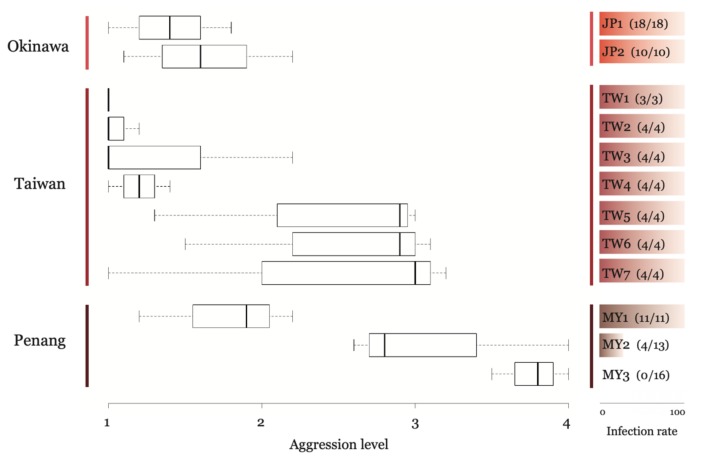
Overview of aggression level and infection rate across the study sites. Numbers in parenthesis denote the number of infected colonies/number of collected colonies in a given site.

We inoculated three virus-free *A*. *gracilipes* colonies by feeding them with a 10% virus-contaminated honey solution, and all the colonies were invariably found positive to TR44839 virus infection as both positive and negative strands of the virus were detected at Day 7 after feeding (Supplementary Figure S1). Hence, we confirmed that the TR44839 virus can be horizontally transmitted among colonies and actively replicate in *A*. *gracilipes*. TR44839 virus occurred in all *A*. *gracilipes* colonies in Okinawa and Taiwan (infection rate 100%), while variations in viral prevalence were observed across the study sites in Penang. Virus-infected colonies in Penang were mostly found in MY1 (100%), whereas the infection rate was lower in sites MY2 (31%), and none in MY3 (0%) (Figure 3).

### 3.2. Colony Boundaries and Aggression Assay

We found significant differences in aggression levels between the three islands (Kruskal–Wallis rank-sum test: χ^2^ = 21.087 df = 2, *p*-Value < 0.001) (Supplementary Figure S2). In general, colonies collected from Okinawa were characterized by the lowest within-site aggression level. Interestingly, individual workers from within-site colony pairs in Taiwan frequently displayed “biting”, but rarely “prolonged aggression”. The highest level of within-site aggression (i.e., level 4) was commonly observed in colony pairs involving colonies collected from the Penang island (Figure 3; Supplementary Table S1). Site differences in aggression behavior were observed in the colonies in Taiwan and Penang (Figure 3). In Taiwan, the within-site aggression level could be divided into two groups, with one group showing little aggression (TW1-TW4) and the other group showing high aggression (TW5-TW7). Mapping the aggression level onto the map revealed a general trend in which aggressive behavior decreased as the latitude increased (Figure 3; Supplementary Figure S2). Moreover, the colonies collected from sites in MY2 and MY3 displayed a high level of aggression, even though the colonies were in close geographic proximity.

### 3.3. Correlation between Infection Status and Aggressive Behavior

**Figure 4:**
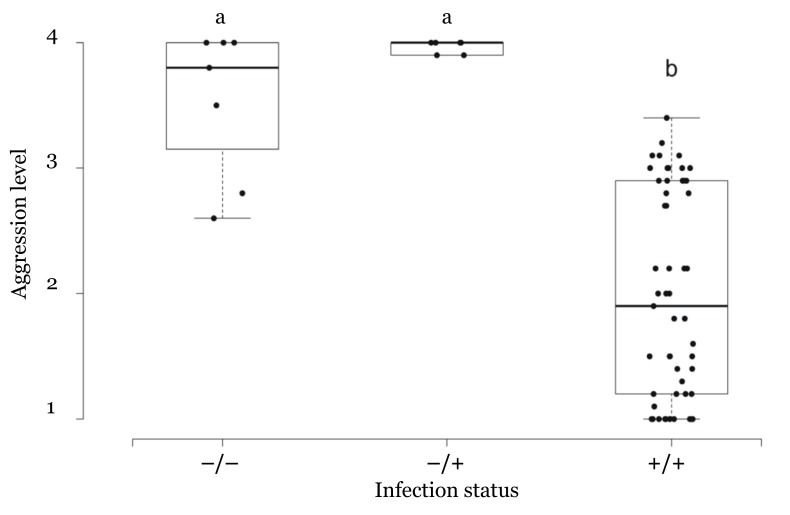
The aggression level of each colony pair in the three categories based on the infection status of interacting workers. (−/−) denotes the pair involving both workers from uninfected colonies; (−/+) denotes the pair involving one infected worker, and one uninfected worker; (+/+) denotes the pair involving both workers from infected colonies. The letters indicate the significant differences between group (*p*-Value < 0.001).

Our analysis showed that aggression levels significantly differed between colony pairs with different infection statuses (Kruskal–Wallis rank-sum test: χ^2^ = 25.63, df = 2, *p*-Value < 0.001). For example, pairs displaying a high level of aggression generally involved one worker that was infection free, while the aggression level significantly decreased when both workers were infected (Figure 4; Supplementary Table S1).

## 4. Discussion

This study reported that the TR44839 virus is widespread in most study sites, particularly in those sites with *A*. *gracilipes* displaying low to moderate intercolonial aggression (i.e., Okinawa and Taiwan), but much less prevalent in sites with ants showing a high level of intercolonial aggression (i.e., Penang). A plausible explanation for the widespread nature of the TR44839 virus is that intensive interactions between colonies may have facilitated the spread of pathogens. The observed pattern in this study is analogous with the pathogen transmission dynamics at the intracolonial level, where higher frequencies of nestmate interactions can readily promote the spread of pathogens within the colony, implying that colony boundaries and levels of intercolonial interactions may contribute to facilitating/preventing viral transmission in *A*. *gracilipes*.

Formation of supercolonies and the reduction of intercolonial aggression were commonly found in introduced populations of several invasive ant species including *A*. *gracilipes* [2,14,24,25], suggesting that such alterations in social structure may be associated with invasiveness of these ant species. However, the vulnerable supercolony hypothesis predicts that recurring intercolonial contact readily offers more opportunities for pathogen transmission than species with colonies displaying a territorial boundary. Our study adds to evidence supporting the hypothesis, as we found a strong link between weak social boundaries (simple colony composition) and epidemics of the TR44839 virus among the colonies in Okinawa and Taiwan. Additional support for pathogen transmission dynamics at population level can be obtained from red imported fire ant. Polygynous fire ant colonies harbor a significantly higher diversity of parasites and pathogens when compared to their monogyne conspecifics, and such differences are attributed to the presence of intensive intercolonial interactions among polygyne colonies, such as resource and worker exchanges between colonies [5,26,27]. Intensive intercolonial interaction as a result of social structure alteration following invasion may also help explain the finding of viruses (if present) generally persisting in a higher prevalence in introduced areas than those in the native ranges [28,29].

A previous study has shown that *A*. *gracilipes* colonies in Penang displayed a high level of intercolonial aggression, and that high mortality was common during the intraspecific aggression assay [16]. Our findings are consistent with this earlier study, and allow us to presume that the population structure of *A*. *gracilipes* in this region is heterogeneous and may harbor multiple localized supercolonies. Interestingly, we found that the intercolonial aggression level appears to decrease as the latitude increases (Figure 3; Supplementary Figure S2), suggesting the presence of a latitudinal gradient of aggression level and possibly population heterogeneity as well. While the origin of *A*. *gracilipes* remains open to debate, our data seem to favor a Southeast Asian origin for *A*. *gracilipes,* because the supercolony structure of this ant in Penang, Malaysia resembles that in native Argentine ants, whose supercolony is generally more localized than populations in their introduced ranges [24,30]. Indeed, emerging evidence has been accumulated to support this ant originating from Southeast Asia. For example, an earlier study by Ito et al. [31] found several incipient colonies of *A*. *gracilipes* that have apparently been initiated by single solitary queens independently in East Java, Indonesia, and the discovery of *A*. *gracilipes* queens adopting such “ancestral” founding strategy may, therefore, imply that this area and possibly other parts of Southeast Asia could be their native range. Large-scale surveys and the development of high-resolution genetic markers are urgently warranted.

## 5. Conclusions

Numerous positive-sense, single-strain RNA viruses have been reported in multiple invasive ant species [17,32,33] with a wide range of effects on their host ants spanning from the reduction of foraging activity [34] and fitness costs [35], to colony mortality [36,37], allowing the utilization of these viruses as biocontrol agents to become feasible and promising [38]. One necessary piece of information to evaluate the biocontrol potential of viruses lies in how these viruses are transmitted between colonies, yet most of the mechanism studies are largely based on a single ant system (i.e., red imported fire ant) and its viruses [39,40]. While other factors may have been involved, the current study has demonstrated that aggression levels among colonies may represent one contributor in shaping viral disease transmission in *A*. *gracilipes*. Future study should examine if intercolonial aggression remains an effective factor associated with virus transmission in other ant-virus systems, as well as the role of other biotic and abiotic factors.

## Supplementary Materials

The following are available online at https://www.mdpi.com/2075-4450/10/12/436/s1, Supplementary Figure S1: Agarose gel electrophoresis for RT-PCR detection of TR44839 virus. RNA of five workers from each of the three additional colonies (colony 1–3, denoted as C1, 2 and 3) was extracted and used as the template for cDNA synthesis using oligo-dT primer and the strand-specific primer. cDNA was then added to the subsequent PCR reaction in which the target viral fragment was amplified. The presence of TR44839 virus is conformed if a 130 bp fragment is amplified by the PCR reaction with template cDNA synthesized using oligo-dT primer (lane 1–3), whereas active replication is evident if a 130 bp fragment is amplified by the PCR reaction with template cDNA synthesized using the strand-specific primer (lane 4–6). M, 100 bp DNA ladder; B, blank; N, negative control; P, positive control. Supplementary Figure S2: Intercolonial aggression level of colony pairs involving two colonies collected from the same site. The numbers of pairwise aggression test are 6, 21 and 9 in Okinawa, Taiwan and Penang, respectively. Different letters indicate the significant differences in aggression level between islands (*p*-Value < 0.001). Supplementary Table S1: Results for within-site and between-site aggression tests.

## References

[1] Eyer P.A., Matsuura K., Vargo E.L., Kobayashi K., Yashiro T., Suehiro W., Himuro C., Yokoi T., Guenard B., Dunn R.R. (2018). Inbreeding tolerance as a pre-adapted trait for invasion success in the invasive ant *Brachyponera chinensis*. Mol. Ecol..

[2] Eyer P.A., McDowell B., Johnson L.N.L., Calcaterra L.A., Fernandez M.B., Shoemaker D., Puckett R.T., Vargo E.L. (2018). Supercolonial structure of invasive populations of the tawny crazy ant *Nylanderia fulva* in the US. BMC Evol. Biol..

[3] King J.R., Tschinkel W.R. (2016). Experimental evidence that dispersal drives ant community assembly in human-altered ecosystems. Ecology.

[4] Van Wilgenburg E., Torres C.W., Tsutsui N.D. (2010). The global expansion of a single ant supercolony. Evol. Appl..

[5] Hoffmann B.D., Hagedorn H. (2014). Quantification of supercolonial traits in the yellow crazy ant, *Anoplolepis gracilipes*. J. Insect. Sci..

[6] Fournier D., Tindo M., Kenne M., Masse P.S.M., Van Bossche V., De Coninck E., Aron S. (2012). Genetic structure, nestmate recognition and behaviour of two cryptic species of the invasive big-headed ant *Pheidole megacephala*. PLoS ONE.

[7] Tragust S., Feldhaar H., Espadaler X., Pedersen J.S. (2015). Rapid increase of the parasitic fungus *Laboulbenia formicarum* in supercolonies of the invasive garden ant *Lasius neglectus*. Biol. Invasions.

[8] Ugelvig L.V., Cremer S. (2012). Effects of social immunity and unicoloniality on host–parasite interactions in invasive insect societies. Funct. Ecol..

[9] Valles S.M., Porter S.D., Choi M.Y., Oi D.H. (2013). Successful transmission of *Solenopsis invicta* virus 3 to *Solenopsis invicta* fire ant colonies in oil, sugar, and cricket bait formulations. J. Invertebr. Pathol..

[10] Dallas T.A., Krkosek M., Drake J.M. (2018). Experimental evidence of a pathogen invasion threshold. R. Soc. Open Sci..

[11] Kavaliers M., Choleris E. (2018). The role of social cognition in parasite and pathogen avoidance. Philos. Trans. R. Soc. Lond. B Biol. Sci..

[12] Altizer S., Nunn C.L., Thrall P.H., Gittleman J.L., Antonovics J., Cunningham A.A., Dobson A.P., Ezenwa V., Jones K.E., Pedersen A.B. (2003). Social organization and parasite risk in mammals: Integrating theory and empirical studies. Annu. Rev. Ecol. Evol. Syst..

[13] Wetterer J.K. (2005). World-wide distribution and potential spread of the long-legged ant, *Anoplolepis gracilipes* (Hymenoptera: Formicidae). Sociobiology.

[14] Abbott K.L. (2005). Supercolonies of the invasive yellow crazy ant, *Anoplolepis gracilipes*, on an oceanic island: Forager activity patterns, density and biomass. Insect. Soc..

[15] Green P.T., O’Dowd D.J., Abbott K.L., Jeffery M., Retallick K., Mac N.R. (2011). Invasional meltdown: Invader-invader mutualism facilitates a secondary invasion. Ecology.

[16] Chong K.F., Lee C.Y. (2010). Inter-and intraspecific aggression in the invasive longlegged ant (Hymenoptera: Formicidae). J. Econ. Entomol..

[17] Cooling M., Gruber M.A.M., Hoffmann B.D., Sébastien A., Lester P.J. (2017). A metatranscriptomic survey of the invasive yellow crazy ant, *Anoplolepis gracilipes*, identifies several potential viral and bacterial pathogens and mutualists. Insect. Soc..

[18] Bonning B.C. (2009). The *Dicistroviridae*: An emerging family of invertebrate viruses. Virol. Sin..

[19] Suarez A.V., Tsutsui N.D., Holway D.A., Case T.J. (1999). Behavioral and genetic differentiation between native and introduced populations of the Argentine ant. Biol. Invasions.

[20] Berville L., Blight O., Renucci M., Hefetz A., Provost E. (2013). A peaceful zone bordering two Argentine ant (*Linepithema humile*) supercolonies. Chemoecology.

[21] Naug D., Camazine S. (2002). The role of colony organization on pathogen transmission in social insects. J. Theor. Biol..

[22] Konrad M., Pull C.D., Metzler S., Seif K., Naderlinger E., Grasse A.V., Cremer S. (2018). Ants avoid superinfections by performing risk-adjusted sanitary care. Proc. Natl. Acad. Sci. USA.

[23] R Core Team (2018). R: A Language and Environment for Statistical Computing.

[24] Giraud T., Pedersen J.S., Keller L. (2002). Evolution of supercolonies: The Argentine ants of southern Europe. Proc. Natl. Acad. Sci. USA.

[25] Krapf P., Russo L., Arthofer W., Most M., Steiner F.M., Schlick-Steiner B.C. (2018). An Alpine ant’s behavioural polymorphism: Monogyny with and without internest aggression in *Tetramorium alpestre*. Ethol. Ecol. Evol..

[26] Oi D.H. (2006). Effect of mono-and polygyne social forms on transmission and spread of a microsporidium in fire ant populations. J. Invertebr. Pathol..

[27] Valles S.M., Oi D.H., Porter S.D. (2010). Seasonal variation and the co-occurrence of four pathogens and a group of parasites among monogyne and polygyne fire ant colonies. Biol. Control.

[28] Yang C.C., Yu Y.C., Valles S.M., Oi D.H., Chen Y.C., Shoemaker D., Wu W.J., Shih C.J. (2010). Loss of microbial (pathogen) infections associated with recent invasions of the red imported fire ant *Solenopsis invicta*. Biol. Invasions.

[29] Sébastien A., Lester P.J., Hall R.J., Wang J., Moore N.E., Gruber M.A.M. (2015). Invasive ants carry novel viruses in their new range and form reservoirs for a honeybee pathogen. Biol. Lett..

[30] Vogel V., Pedersen J.S., d’Ettorre P., Lehmann L., Keller L. (2009). Dynamics and genetic structure of Argentine ant supercolonies in their native range. Evolution.

[31] Ito F., Asfiya W., Kojima J. (2016). Discovery of independent-founding solitary queens in the yellow crazy ant *Anoplolepis gracilipes* in East Java, Indonesia (Hymenoptera: Formicidae). Entomol. Sci..

[32] Valles S.M. (2012). Positive-strand RNA viruses infecting the red imported fire ant, *Solenopsis invicta*. Psyche.

[33] Viljakainen L., Holmberg I., Abril S., Jurvansuu J. (2018). Viruses of invasive Argentine ants from the European Main supercolony: Characterization, interactions and evolution. J. Gen. Virol..

[34] Hsu H.W., Chiu M.C., Shoemaker D., Yang C.S. (2018). Viral infections in fire ants lead to reduced foraging activity and dietary changes. Sci. Rep..

[35] Manfredini F., Shoemaker D., Grozinger C.M. (2016). Dynamic changes in host-virus interactions associated with colony founding and social environment in fire ant queens (*Solenopsis invicta*). Ecol. Evol..

[36] Valles S.M., Hashimoto Y. (2009). Isolation and characterization of *Solenopsis invicta* virus 3, a new positive-strand RNA virus infecting the red imported fire ant, *Solenopsis invicta*. Virology.

[37] Valles S.M., Strong C.A., Dang P.M., Hunter W.B., Pereira R.M., Oi D.H., Shapiro A.M., Williams D.F. (2004). A picorna-like virus from the red imported fire ant, *Solenopsis invicta*: Initial discovery, genome sequence, and characterization. Virology.

[38] Oi D., Valles S., Porter S., Cavanaugh C., White G., Henke J. (2019). Introduction of fire ant biological control agents into the Coachella Valley of California. Fla. Entomol..

[39] Hashimoto Y., Valles S.M. (2007). *Solenopsis invicta* virus-1 tissue tropism and intra-colony infection rate in the red imported fire ant: A quantitative PCR-based study. J. Invertebr. Pathol..

[40] Chen Y., Evans J., Feldlaufer M. (2006). Horizontal and vertical transmission of viruses in the honey bee, *Apis mellifera*. J. Invertebr. Pathol..

