# Supplementary material for: The Association between Virus Prevalence and Intercolonial Aggression Levels in the Yellow Crazy Ant, Anoplolepis Gracilipes (Jerdon)
Source: Insects. 2019 Dec 4;10(12):436. doi: 10.3390/insects10120436 (PMC6956197; doi:10.3390/insects10120436)
Supplement: Supplementary file 1 [file insects-10-00436-s001.pdf]

Supplementary Figures

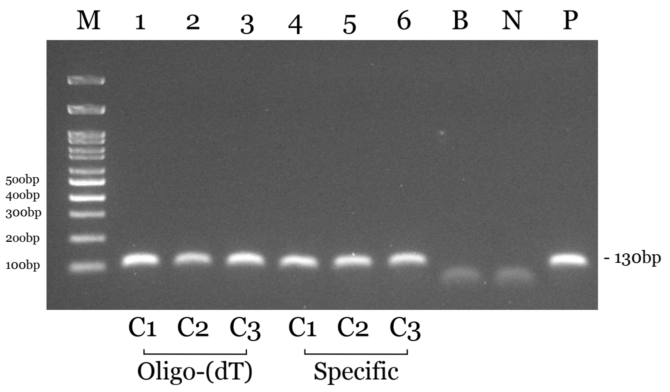

Supplementary Figure 1. Agarose gel electrophoresis for RT-PCR detection of TR44839 virus. RNA of five workers from each of the three additional colonies (colony 1-3, denoted as C1, 2 and 3) was extracted and used as the template for cDNA synthesis using oligo-dT primer and the strand-specific primer. cDNA was then added to the subsequent PCR reaction in which the target viral fragment was amplified. The presence of TR44839 virus is conformed if a 130 bp fragment is amplified by the PCR reaction with template cDNA synthesized using oligo-dT primer (lane 1-3), whereas active replication is evident if a 130 bp fragment is amplified by the PCR reaction with template cDNA synthesized using the strand-specific primer (lane 4-6). M, 100 bp DNA ladder; B, blank; N, negative control; P, positive control.

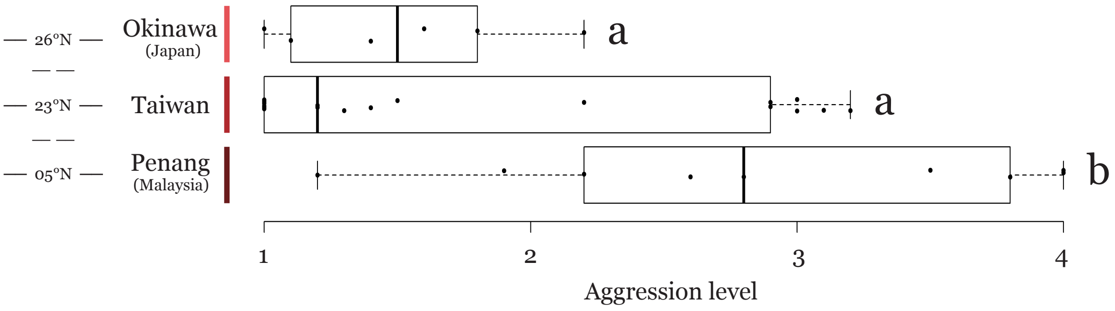

Supplementary Figure 2. Intercolonial aggression level of colony pairs involving two colonies collected from the same site. Sample size for the aggression test is 6, 21 and 9 in Okinawa, Taiwan and Penang, respectively. Different letters indicate the significant differences in aggression level between islands (p-value<0.001).

Supplementary Table 1 Results for within-site and between-site aggression tests

Whitin-site colony pair

Region	Site	Pair	Distance (Km)	Infection status	Aggression assay (10 replicates)										
					1	2	3	4	5	6	7	8	9	10	Average
**Okinawa**	**1**	**1-1 vs 1-3**	0.152	(+ / +)	2	2	1	1	3	3	2	2	1	1	1.8
**Okinawa**	**1**	**1-2 vs 1-3**	0.167	(+ / +)	1	1	1	1	1	1	1	1	1	1	1
**Okinawa**	**1**	**1-1 vs 1-2**	0.117	(+ / +)	3	1	1	3	1	1	1	1	1	1	1.4
**Okinawa**	**2**	**2-1 vs 2-3**	0.149	(+ / +)	1	1	1	1	1	1	1	2	1	1	1.1
**Okinawa**	**2**	**2-2 vs 2-3**	0.12	(+ / +)	2	1	2	1	2	3	1	1	2	1	1.6
**Okinawa**	**2**	**2-1 vs 2-2**	0.121	(+ / +)	2	1	1	1	3	2	3	3	3	3	2.2
**Taiwan**	**1**	**1-1 vs 1-3**	0.104	(+ / +)	1	1	1	1	1	1	1	1	1	1	1
**Taiwan**	**1**	**1-2 vs 1-3**	0.103	(+ / +)	2	1	1	1	2	1	1	1	1	1	1.2
**Taiwan**	**1**	**1-1 vs 1-2**	0.16	(+ / +)	1	1	1	1	1	1	1	1	1	1	1
**Taiwan**	**2**	**2-1 vs 2-3**	0.18	(+ / +)	1	1	1	1	1	1	1	1	1	1	1
**Taiwan**	**2**	**2-2 vs 2-3**	0.123	(+ / +)	1	3	3	3	3	1	3	1	1	3	2.2
**Taiwan**	**2**	**2-1 vs 2-2**	0.128	(+ / +)	1	1	1	1	1	1	1	1	1	1	1
**Taiwan**	**3**	**3-1 vs 3-3**	0.149	(+ / +)	1	1	1	1	1	1	1	1	1	1	1
**Taiwan**	**3**	**3-2 vs 3-3**	0.133	(+ / +)	1	1	2	1	2	3	1	1	1	1	1.4
**Taiwan**	**3**	**3-1 vs 3-2**	0.11	(+ / +)	1	1	2	1	1	2	1	1	1	1	1.2
**Taiwan**	**4**	**4-1 vs 4-3**	0.15	(+ / +)	3	3	3	3	3	3	3	3	3	3	3
**Taiwan**	**4**	**4-2 vs 4-3**	0.29	(+ / +)	4	3	3	3	3	4	3	3	3	3	3.2
**Taiwan**	**4**	**4-1 vs 4-2**	0.444	(+ / +)	1	1	1	1	1	1	1	1	1	1	1
**Taiwan**	**5**	**5-1 vs 5-3**	0.528	(+ / +)	1	1	1	1	1	1	3	1	1	2	1.3
**Taiwan**	**5**	**5-2 vs 5-3**	0.42	(+ / +)	3	3	3	3	3	3	3	3	3	3	3
**Taiwan**	**5**	**5-1 vs 5-2**	0.273	(+ / +)	3	3	3	3	3	3	2	3	3	3	2.9
**Taiwan**	**6**	**6-1 vs 6-3**	2.32	(+ / +)	1	1	1	1	1	1	1	1	1	1	1
**Taiwan**	**6**	**6-2 vs 6-3**	3.69	(+ / +)	1	1	1	1	1	1	1	1	1	1	1
**Taiwan**	**6**	**6-1 vs 6-2**	1.74	(+ / +)	1	1	1	1	1	1	1	1	1	1	1
**Taiwan**	**7**	**7-1 vs 7-3**	7.61	(+ / +)	3	3	3	3	3	3	3	3	2	3	2.9
**Taiwan**	**7**	**7-2 vs 7-3**	6.48	(+ / +)	3	3	3	3	3	3	3	4	3	3	3.1
**Taiwan**	**7**	**7-1 vs 7-2**	1.78	(+ / +)	1	1	1	2	1	3	1	3	1	1	1.5
**Penang**	**1**	**1-1 vs 1-3**	0.606	(+ / −)	4	4	4	4	4	4	4	4	4	4	4
**Penang**	**1**	**1-2 vs 1-3**	0.579	(− / −)	4	3	3	4	4	3	4	3	3	4	3.5
**Penang**	**1**	**1-1 vs 1-2**	0.157	(− / −)	4	4	4	4	3	4	4	3	4	4	3.8
**Penang**	**2**	**2-1 vs 2-3**	1.25	(− / −)	3	3	3	2	3	2	3	3	3	3	2.8
**Penang**	**2**	**2-2 vs 2-3**	0.156	(− / −)	3	3	3	2	3	3	1	2	3	3	2.6
**Penang**	**2**	**2-1 vs 2-2**	1.35	(+ / −)	4	4	4	4	4	4	4	4	4	4	4

**Penang**	**3**	**3-1 vs 3-3**	0.227	(+ / +)	3	3	3	3	1	1	1	1	3	3	2.2
**Penang**	**3**	**3-2 vs 3-3**	0.12	(+ / +)	1	1	2	1	3	3	3	3	1	1	1.9
**Penang**	**3**	**3-1 vs 3-2**	0.256	(+ / +)	1	1	1	1	3	1	1	1	1	1	1.2

Between-site colony pair

Region	Site	Pair	Distance (Km)	Infection status	Aggression assay (10 replicates)										
					1	2	3	4	5	6	7	8	9	10	Average
**Okinawa**	**1 vs 2**	**1-3 vs 2-3**	11.5	(+ / +)	1	1	2	1	3	3	3	3	2	1	2
**Okinawa**	**1 vs 2**	**1-1 vs 2-3**	12.3	(+ / +)	1	1	2	2	3	1	1	1	2	1	1.5
**Okinawa**	**1 vs 2**	**1-3 vs 2-2**	11.7	(+ / +)	2	3	3	1	1	1	1	1	1	1	1.5
**Okinawa**	**1 vs 2**	**1-3 vs 2-1**	11.5	(+ / +)	1	1	1	1	3	3	3	3	1	1	1.8
**Okinawa**	**1 vs 2**	**1-2 vs 2-2**	11.4	(+ / +)	1	1	1	1	3	3	2	1	1	1	1.5
**Okinawa**	**1 vs 2**	**1-2 vs 2-3**	11.2	(+ / +)	3	1	1	1	1	1	1	1	1	1	1.2
**Taiwan**	**1 vs 2**	**1-1 vs 2-3**	0.531	(+ / +)	3	3	3	3	3	3	3	3	4	3	3.1
**Taiwan**	**1 vs 2**	**1-1 vs 2-1**	0.527	(+ / +)	3	3	3	3	3	3	1	3	3	3	2.8
**Taiwan**	**1 vs 2**	**1-3 vs 2-3**	0.47	(+ / +)	3	3	3	2	3	3	3	3	3	3	2.9
**Taiwan**	**1 vs 2**	**1-1 vs 2-2**	0.49	(+ / +)	3	3	4	3	3	3	1	3	1	3	2.7
**Taiwan**	**1 vs 3**	**1-1 vs 3-1**	0.168	(+ / +)	1	1	1	1	1	1	1	1	1	1	1
**Taiwan**	**1 vs 3**	**1-3 vs 3-3**	0.126	(+ / +)	1	3	3	3	3	1	1	1	1	3	2
**Taiwan**	**1 vs 5**	**1-1 vs 5-2**	177	(+ / +)	3	3	3	3	2	3	2	4	3	4	3
**Taiwan**	**1 vs 5**	**1-1 vs 5-3**	177	(+ / +)	3	3	3	3	3	3	3	3	4	3	3.1
**Taiwan**	**2 vs 3**	**2-2 vs 3-3**	0.543	(+ / +)	3	1	3	3	4	3	1	4	3	4	2.9
**Taiwan**	**2 vs 3**	**2-3 vs 3-3**	0.528	(+ / +)	4	3	3	3	3	1	3	3	3	3	2.9
**Taiwan**	**2 vs 4**	**2-1 vs 4-3**	7.45	(+ / +)	3	3	3	3	3	3	3	1	3	3	2.8
**Taiwan**	**2 vs 4**	**2-2 vs 4-3**	7.44	(+ / +)	1	3	3	3	3	1	1	3	1	3	2.2
**Taiwan**	**2 vs 4**	**2-3 vs 4-3**	7.45	(+ / +)	1	3	3	3	3	1	1	1	1	3	2
**Taiwan**	**3 vs 4**	**3-3 vs 4-3**	7.71	(+ / +)	1	3	3	3	3	3	3	2	3	3	2.7
**Taiwan**	**4 vs 5**	**4-3 vs 5-2**	174	(+ / +)	3	3	2	3	4	4	2	3	3	3	3
**Taiwan**	**4 vs 5**	**4-3 vs 5-3**	174	(+ / +)	4	4	3	3	4	3	4	3	3	3	3.4
**Taiwan**	**6 vs 7**	**6-2 vs 7-3**	53	(+ / +)	3	3	3	3	3	3	3	3	3	3	3
**Penang**	**1 vs 2**	**1-2 vs 2-3**	3.29	(+ / −)	4	4	4	4	4	4	4	4	4	4	4
**Penang**	**1 vs 3**	**1-1 vs 3-3**	17	(+ / −)	4	3	4	4	4	4	4	4	4	4	3.9
**Penang**	**2 vs 3**	**2-1 vs 3-1**	16.4	(+ / −)	4	4	4	4	4	4	4	4	4	4	4
**Penang**	**1 vs 2**	**1-3 vs 2-1**	3.12	(− / −)	4	4	4	4	4	4	4	4	4	4	4
**Penang**	**2 vs 3**	**2-1 vs 3-2**	16.2	(+ / −)	3	4	4	4	4	4	4	4	4	4	3.9
**Penang**	**1 vs 3**	**1-3 vs 3-3**	16.7	(+ / −)	4	4	4	4	4	4	4	4	4	4	4
**Penang**	**1 vs 2**	**1-1 vs 2-2**	30.7	(− / −)	4	4	4	4	4	4	4	4	4	4	4
